# Synthesis of BiOI-TiO_2_ Composite Nanoparticles by Microemulsion Method and Study on Their Photocatalytic Activities

**DOI:** 10.1155/2014/647040

**Published:** 2014-01-16

**Authors:** Yunfang Chen, Xiaoxin Xu, Jianzhang Fang, Guangying Zhou, Zhang Liu, Shuxing Wu, Weicheng Xu, Jinhui Chu, Ximiao Zhu

**Affiliations:** School of Chemistry and Environment, South China Normal University, Guangzhou, Guangdong 510006, China

## Abstract

This study was conducted to synthesize a series of nanosized BiOI-TiO_2_ catalysts to photodegrade Bisphenol A solution. The BiOI-TiO_2_ nanoparticles were synthesized in the reverse microemulsions, consisting of cyclohexane, Triton X-100, n-hexanol, and aqueous salt solutions. The synthesized particles were characterized by X-ray diffraction (XRD), Brunauer-Emmett-Teller (BET) surface analyzer, Fourier transform-infrared spectroscopy (FT-IR), ultraviolet-visible light (UV-Vis) absorption spectra and transmission electron microscope (TEM). The photodegradation of Bisphenol A (BPA) in aqueous suspension under visible light irradiation was investigated to explore the feasibility of using the photocatalytic method to treat BPA wastewater. The effects of different molar ratios of BiOI to TiO_2_ on the photocatalytic activity were discussed. The experimental results revealed that the photocatalytic effect of the BiOI-TiO_2_ particles was superior to the commercial P25 TiO_2_. The BPA degradation could be approached by a pseudo-first-order rate expression. The observed reaction rate constant (*k*_obs_) was related to nanoparticles dosage and initial solution pH.

## 1. Introduction

Over the past several years, heterogeneous photocatalysis by semiconductors provides an economic and ecological method for the remediation of contaminated water and air. Due to its biological and chemical inertness, nontoxicity, and long-term stability [1], TiO_2_ has received much attention as wasted-water treatment. The main shortcoming of anatase TiO_2_, however, is that it only absorbs ultraviolet light no longer than 387.5 nm, which only occupies about 4% of sunlight [2, 3]. Therefore, much work on preparing TiO_2_ photocatalysts with visible light responsibility, such as doping TiO_2_ with transition metals [4], noble metals [5, 6], rare-earth metals [7], and anions [8], has been reported. Up to present, a large number of coupled polycrystalline or colloidal semiconductors [9–14], such as SiO_2_-TiO_2_, and CdS-TiO_2_, ZnO-TiO_2_, SnO_2_-TiO_2_, ZrO_2_-TiO_2_, have been prepared. Among them, BiOI–TiO_2_ compounds exhibit attractive photocatalytic activity on the degradation of organic pollutants under visible-light irradiation due to the fact that BiOI has the estimated band gap of about 1.77 eV which can be excited by visible light irradiation. BiOI-based composite materials have been prepared, such as AgI/BiOI [15, 16]. Motivated by these facts, we are interested in the BiOI-TiO_2_ system.

Nowadays, the research has demonstrated that Bisphenol A [2,2-bis(4-hydroxyphenyl)propane, BPA] is a representative endocrine disrupter that can cause various diseases [17, 18]. However, Bisphenol A (BPA) has been commonly used as raw material for epoxy and polycarbonate resins, such as baby bottles, water bottles, food cans, and flame retardants [19–21]. BPA has been found in food, drinks, indoor and outdoor air, dust, and soil. In previous studies, BPA has been detected at the maximum concentration of 17.2 mg/L in hazardous waste landfill leachates [22]. Traditional methods to remove recalcitrant organic chemicals from effluents include the use of adsorbents, chemical oxidation, biodegradation, and advanced oxidation processes [23–26]. Among these methods, photocatalytic oxidation is one of the most promising technologies due to its high degradation efficiency and utilizes sunlight as energy source.

The major objectives of this work are (1) to prepare a series of BiOI-TiO_2_ nanosized particles by hydrolysis of tetrabutyl titanate and bismuth nitrate hydrate in a microemulsion system under room temperature and ambient pressure and (2) to degrade Bisphenol A (BPA) by the obtained BiOI-TiO_2_ nanosized particles and study the impacts of different reaction factors. The synthesis and characterization of bismuth oxyiodine/titanium dioxide hybrid nanoparticles have been reported by our previous work [27]; this work was focused on the degradation of BPA.

## 2. Materials and Methods

### 2.1. Preparations of Photocatalysts

**Figure 1:**
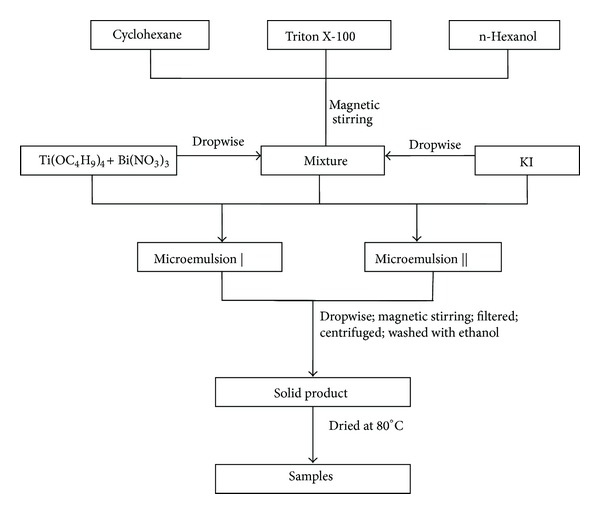
Flow chart of nanosized BiOI-TiO_2_ particles synthesized by microemulsion.

In a typical procedure, as shown in Figure 1, Triton X-100 (chemically pure, CP) used as the surfactant, cyclohexane (analytically pure, AP) as the oil phase, and *n*-hexanol (CP) as the cosurfactant were mixed at quality ratio of 15 : 6 : 4 under magnetic stirring at room temperature. One aqueous phase was bismuth nitrate (AP) and tetrabutyl titanate (AP) dissolved in dilute nitric acid. The other aqueous phase was the potassium iodide (AP) dissolved in dilute ammonia hydroxide solution. Then, they were dropped into the aforementioned suspensions, respectively, until they became transparent. After that, the microemulsion containing KI was added into the microemulsion containing Bi^3+^. The microemulsions were formed, confirmed by the observed Tyndall effect [28]. The resultant suspension was stirred for 3 h and the reaction solution was centrifuged at 3000 rpm. The precipitate was collected and washed with distilled water and anhydrous ethanol for several times to remove any possible organic compounds and surfactants and then dried in an oven at 80°C for 24 h. The obtained precursors were calcined for 3 h at 300°C, and then the final products were milled before characterization. Samples were labeled as *x*% BiOI-TiO_2_ where *x*% is the molar ratio of BiOI to TiO_2_.

### 2.2. Characterization of Photocatalysts

XRD patterns were recorded on a Dan-dong Aolong/Y-2000 X-ray diffractometer (Dan Dong, China) with Cu Ka radiation (*k* = 0.15406 nm). The operating voltage was set at 40 kV, and the current was 40 mA. FT-IR spectra were recorded using a Shimadzu IRPrestige-21 Fourier transform spectrometer (Japan) by blending the sample into a KBr pellet. The measurement of specific surface area of samples was performed using nitrogen adsorption isotherm by specific surface area analyzer (ASAP2020M, Micromeritics Instrument Corp., USA). The UV-Vis light absorption spectra were obtained from a Hitachi UV-3010 spectrophotometer. The particle size and morphology were observed on a JEOL JEM-2010 (HR) transmission electron microscope.

### 2.3. Photocatalytic Studies

The photocatalytic activity of BiOI-TiO_2_ composite semiconductor was evaluated by degradation of BPA in aqueous solution, while commercial P25 powders and mechanically mixed counterpart 75% BiOI + TiO_2_ samples were used for comparison. For all photocatalytic experiments, a cylindrical glass was used as the reactor, which was filled with 200 mL of an aqueous suspension containing photocatalysts and 20 mg·L^−1^ of BPA. Then, the solution was vertically irradiated from the top by a 250 W halogen lamp which provided artificial solar light. The 250 W halogen lamp equipped with an ultraviolet cutoff filter (*λg*> 400 nm) to provide visible light was used as the light source. The illumination distance was about 10 cm. Before irradiation, the suspension was stirred for 15 minutes in the dark to achieve an adsorption-desorption balance on the catalyst surface. After that, about 2 mL of the suspension continually was taken from the reaction cell at given time intervals. Then the sample was filtered immediately through 0.45 *μ*m membrane filters for HPLC analysis. At the end of the reaction, the samples accounted for less than 5% of the volume of solution. Thus, opposite impacts of the changes of the volume were negligible.

### 2.4. Analytical Methods

Bisphenol A (BPA) concentrations were measured by high performance liquid chromatography (HLPC, Shimadzu, Japan) equipped with a UV detector (SPD-10AV) and a C18 column (250 mm × 4.6 mm). The detection wavelength was set at 273 nm. The mobile phase was 70% methanol (HPLC grade) and 30% water at a flow rate of 0.8 mL/min.

## 3. Results and Discussion

### 3.1. Characterization of BiOI-TiO_2_ Composite Particles

**Figure 2:**
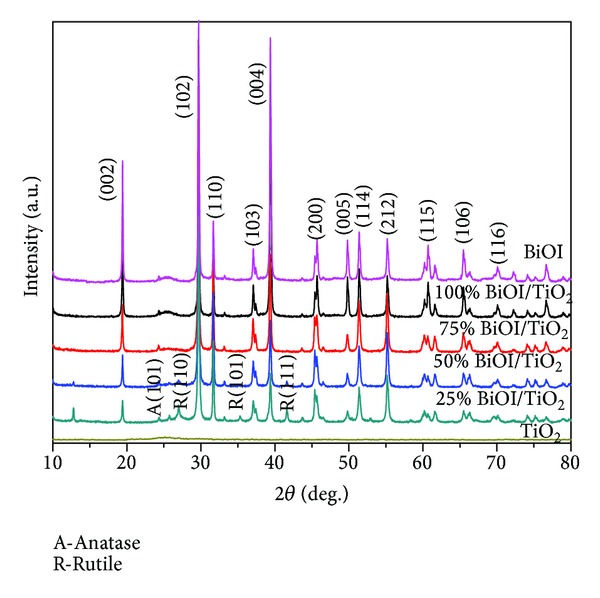
XRD pattern of nanoscale particles.

The X-ray powder diffraction analysis (Figure 2) showed that the as-prepared powders were a mixture phase of BiOI, anatase TiO_2_, and rutile TiO_2_. The BiOI-TiO_2_ samples displayed diffraction peaks around 2*θ* of 29.5, 31.7, which could be indexed to the characteristic peaks of BiOI (JCPDS file no. 10-0445). The XRD peaks at around 25.2° in the spectrum of TiO_2_ were identified as the anatase form, whereas the XRD peaks at 27.4° were taken as the rutile form. With the decrease of TiO_2_ component in BiOI-TiO_2_ composites, the intensity of the peak at 31.7°, which was identified as the main peak of the remnant BiOI phase, was gradually increased. The peak at 25.2° and 27.4° inherent from the TiO_2_ phase was decreased, indicating that the presence of BiOI could inhibit the crystal growth of TiO_2_. The average crystallite size was calculated according to the Scherrer equation:

(1) d=kλβcos θ ,

where *k* is a constant, *λ* is the wavelength of the X-rays, *θ* is the maximum angle of the peak, and *β* is the width of the peak at half height.

The crystallite size of the main peak (BiOI) determined by Scherrer's equation was estimated to be 5–10 nm.

The BET surface areas of the prepared photocatalysts were given in Table 1. It was found that the powder contained small mesopores (3 nm) and large mesopores with maximum pore diameters of 37 nm, determined by using the Barrett-Joyner-Halenda (BJH) method. As shown in Table 1, the BiOI had a poor surface area of 12.2 m^2^g^−1^, while the surface areas of 25% BiOI-TiO_2_, 50% BiOI-TiO_2_, 75% BiOI-TiO_2_, and 100% BiOI–TiO_2_ were 145.3, 101.1, 51.9, and 40.7 m^2^g^−1^, respectively. The results also showed that the *A*_BET_ and *V*_BJH_ decreased with the increase of BiOI ratio in the BiOI-TiO_2_ coupling particles.

**Figure 3:**
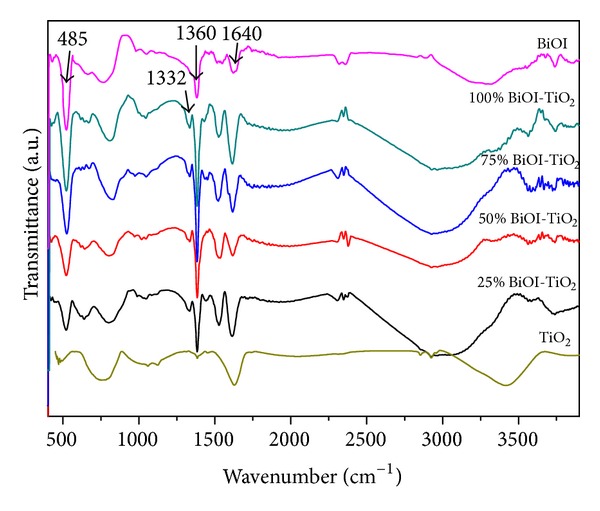
FT-IR spectra of pure TiO_2_, BiOI, and as-prepared BiOI-TiO_2_ nanoparticles.

The surface hydroxyl groups on photocatalysts were recognized to play an important role in the photocatalytic reaction since they could inhibit the recombination of photogeneration charges and interact with photogenerated holes to produce active oxygen species. What is more important is to produce an OH-radical by reaction with a photogenerated hole and in this way provide an important oxidant. The FT-IR transmittance spectra of the samples were shown in Figure 3. The band at about 512 cm^−1^ could be attributed to the vibration of Ti–O–Ti. The strong peak at 1640 cm^−1^ was ascribed to the bending vibration absorption of chemically adsorbed water, and the peaks at 3000 cm^−1^ −3420^−1^ cm were attributed to the stretching vibration absorption of hydroxyl function groups (TiO_2_–OH bonds). It was believed that such groups arose from the hydrolysis reaction in the microemulsion process [29]. The band centered at 1332 cm^−1^ was assigned to bending vibrations of C–H bond in the species linking-Ti-O-Ti-structural network. The intensity of the absorption appeared at 485 cm^−1^, which could be assigned to the Bi-O-Ti stretching [30], increased obviously with the enhancement of BiOI content in BiOI-TiO_2_ heterojunctions.

**Figure 4:**
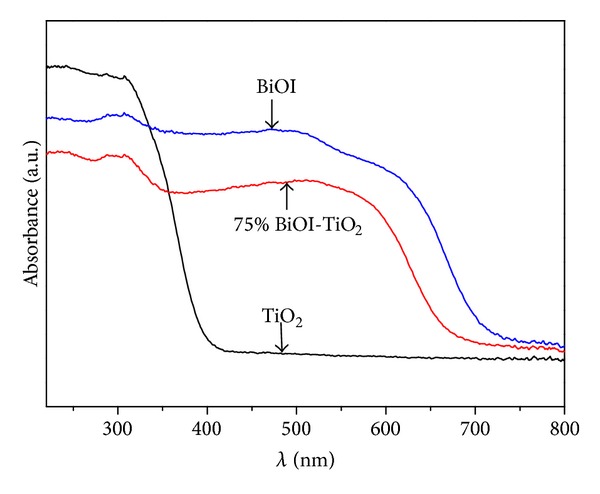
UV-Vis diffuse reflectance spectra of TiO_2_, BiOI, and BiOI-TiO_2_ nanoparticles.

Figure 4 showed the UV–Vis spectra of pure TiO_2_ powders, BiOI powders, and the 75% BiOI-TiO_2_ composite particles, which was proved the most efficient of the series of x% BiOI-TiO_2_ samples. This figure illustrated that The UV absorption edge had a monotonic red with the join of BiOI, which suggested that the BiOI addition led to absorption increase in the visible region. The pure TiO_2_ had high absorption in the UV region but relatively low absorption in visible light region. In contrast, the BiOI powders had the higher absorption in UV region than pure TiO_2_. Obviously, the BiOI-TiO_2_ particles prepared by microemulsion method resulted in the shift of the absorbance region toward longer wavelength. The band gap could be determined by the following equation [31]:

(2) αhν=A(hν−Eg)n2,

where *α* is absorption coefficient, *ν* is light frequency, *A* is proportionality constant, and Eg is band gap.

The band gaps of the samples estimated from the equation were from 1.9 eV(BiOI) to 3.2 eV(TiO_2_). This piece of information suggested that the synthetized BiOI-TiO_2_ had the smaller band gap than TiO_2_ and had the better photocatalytic activities in visible region.

**Figure 5:**
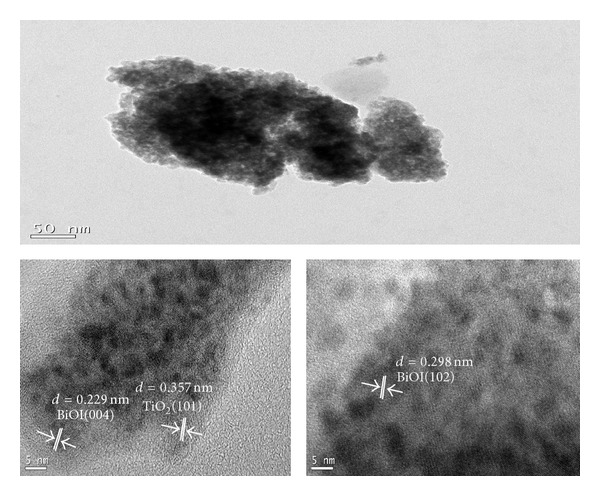
TEM image of 75% BiOI-TiO_2_ nanoparticles.

Figure 5 showed the typical TEM images of 75% BiOI-TiO_2_. It was observed that 75% BiOI-TiO_2_ particles synthesized in microemulsion were sheet structure. Some nanoparticles with sizes of several nanometers were attached on the surface, which were thought to be TiO_2_. The TEM pattern showed that the TiO_2_ nanoparticles were laid on the BiOI flakes. This might suggest why XRD diffraction patterns of TiO_2_ decreased obviously with the increased presence of BiOI.

### 3.2. Effect of BiOI-TiO_2_ Molar Ratio on the Degradation

**Figure 6:**
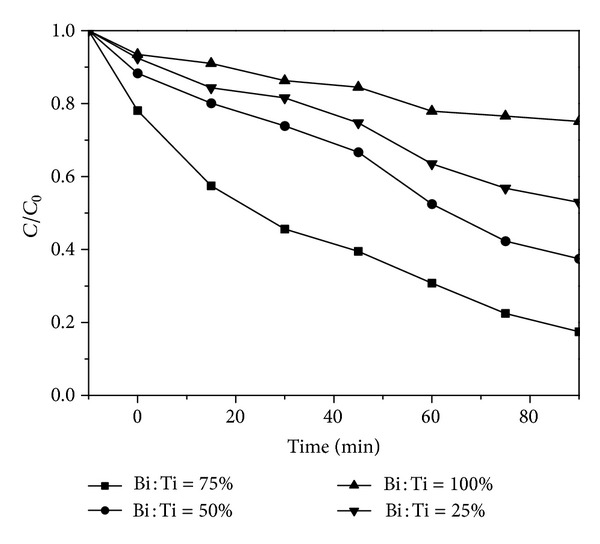
The effects of different ratio rate of BiOI to TiO_2_ on the photocatalytic degradation of BPA. Catalyst dosage = 0.5 g/L, initial pH = 5.8, and BPA concentration = 20 mg/L.

In order to study the effects of different BiOI/TiO_2_ molar ratios on the photocatalytic degradation, the samples with Bi/Ti = 25%, 50%, 75%, and 100% were used for degradation of BPA.  Figure 6 showed the effects of different ratio rates of BiOI to TiO_2_ on the photocatalytic degradation of BPA. It was well known that pure TiO_2_ exhibited an inability for BPA degradation, for it almost cannot absorb visible light. Also to BiOI, it was said that pure BiOI had a narrow band gap and electronic hole so that it would be recovered quickly upon visible-light irradiation. The synthesized BiOI-TiO_2_ might overcome these shortcomings. As the loading amounts of BiOI increased from 25% to 100%, it could be seen that the degradation percentages of BPA were 47.1%, 62.5%, 82.5% and 24.9%, respectively. Therefore, the results indicated that the as-prepared BiOI-TiO_2_ composite materials had dramatically improved the photocatalytic degradation of BPA.

The photocatalytic degradation of BPA was observed to follow a first-order kinetic reaction:

(3) lnctc0=−kobst,

where *c*_*t*_ is the concentration of BPA at selected times (mg/L); *c*_0_ is the initial BPA concentration (mg/L); *k*_obs_ is the observed rate constant (min^−1^).

The kinetic data under different experimental conditions were listed in Table 2. The *k*_obs_ of the samples with Bi/Ti = 25%, 50%, 75%, and 100% were 0.097, 0.150, 0.241, and 0.040 min^−1^, respectively. Obviously, the samples with Bi/Ti = 75% had the better photocatalytic effects than other as-prepared samples. However, further research is needed to explain why 75% BiOI-TiO_2_ sample exhibited the highest photocatalytic activity.

### 3.3. Effect of Catalysts Dosage

**Figure 7:**
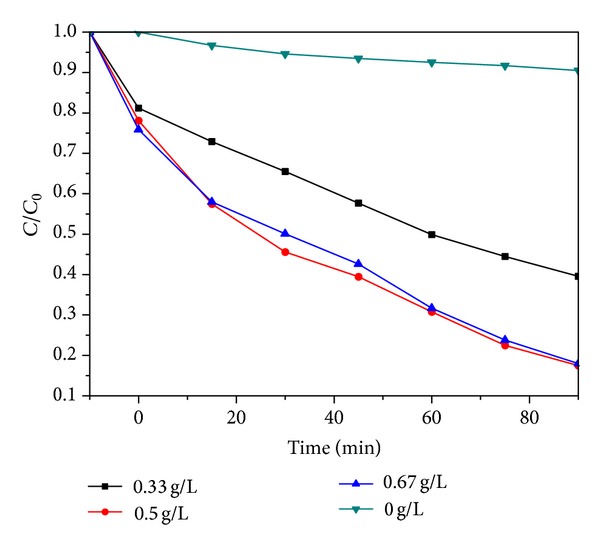
Effect of 75% BiOI-TiO_2_ dosage on the degradation of BPA. Initial pH = 5.8,  BPA concentration = 20 mg/L.

The degradation efficiency of BPA by 75% BiOI/TiO_2_ with different catalysts dosages was illustrated in Figure 7. When the BiOI-TiO_2_ composite material dosages set at 0–0.500 g/L, the removal efficiency was significantly enhanced. Because photocatalytic reaction occurred on the surface of BiOI-TiO_2_ composite material and the available surface area, adsorption and reaction sites were increased with increasing concentration of nanoparticles. When the dosage of the catalysts was increased continuously (above 0.500 g/L), however, the photodegradation activity was decreased. It was commonly accepted that too much dark red nanoparticles were suspended in Bisphenol A solution, which could reject the generation of e^−^ and h^+^ due to the increase of light scattering [32]. Furthermore, the proportion of incident light that was absorbed by the catalyst increased.

The kinetic data under the different catalysts dosages was summarized in Table 2. The observed rate constants were 0.015–0.241 min^−1^ and reached thier highest at catalysts dosage of 0.500 g/L. Based on the results, the appropriate dosage of BiOI/TiO_2_ composite material in the level of 0.500 g/L was chosen for subsequent BPA degradation.

### 3.4. Effect of Initial PH

In heterogeneous catalyst, the active sites on the surface of most semiconductors may affect the concentration of hydrogen ion or hydroxide ion in aqueous solution [33]. Therefore, pH of the reaction solution may affect the catalytic activity of the photocatalyst. To investigate the interaction of the effect, degradation of BPA was examined at a series of pH values. In this study, BPA solution was adjusted to different initial pH values by diluting hydrochloric acid or sodium hydroxide, without pH adjusting during the reaction.

**Figure 8:**
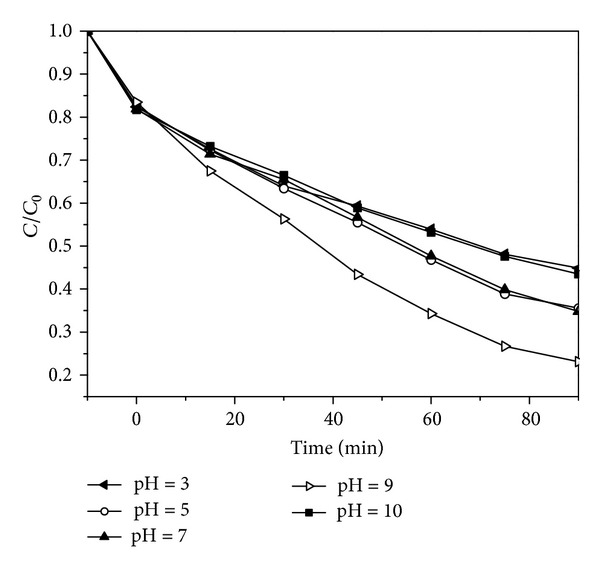
Effect of initial pH on BPA removal rate. Catalyst dosage = 0.5 g/L, BPA concentration = 20 mg/L.

Figure 8 presented the influence of initial pH on the photocatalytic degradation of BPA on 75% BiOI-TiO_2_ sample. Obviously, the best degradation efficiency was reached at neutral pH, while the low photodegradation rates were recorded in highly acidic and alkaline conditions. It was already known that photocatalytic efficiency was significantly decreased in the acidic condition by inhibiting the generation of OH radical [34–36]. It was noticed that, while there is no pH change in the solution, the initial pH was about 5.8 (at neutral pH range). The results indicated that the best condition for BPA degradation could be neutral pH and no pH adjustment on the reaction solution was needed.

### 3.5. Comparison of BPA Degradation by Commercial P25 and BIOI + TiO_2_

**Figure 9:**
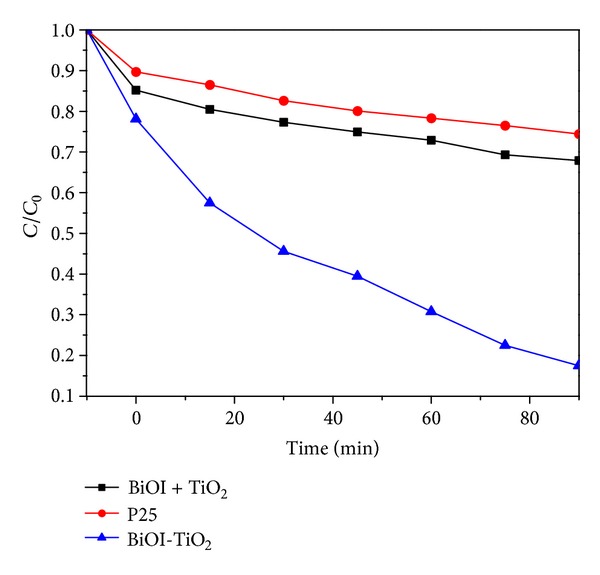
Comparison of Bisphenol A degradation by commercial P25, 75% BiOI + TiO_2_, and synthesized BiOI-TiO_2_.

The degradations by commercial P25 and mechanically mixed counterpart 75% BiOI + TiO_2_ samples were performed under the benchmark experiment conditions: initial BPA concentration of 20 mg/L, initial pH of 5.8, and catalysts dosage of 0.5 g/L. The results were illustrated in Figure 9.

The results indicated that both commercial P25 and mechanically mixed counterpart 75% BIOI + TiO_2_ could not effectively eliminate BPA. The removal efficiencies were only 32.1% and 25.6%, respectively. The corresponding rate constants *k*_obs_ were 0.037 and 0.031, which were much lower than the samples we prepared. The BiOI prepared in this work with narrow band gap energy (1.79 eV) could be easily excited by visible light and could induce the generation of photoelectrons and holes, but these photoelectrons and holes might also recombine rapidly. TiO_2_ could only absorb ultraviolet light no longer than 387.5 nm. Hence, mechanically mixed counterpart sample BiOI + TiO_2_ and commercial P25 particles showed poor photocatalytic activity under visible-light irradiation.

The synthesized BiOI-TiO_2_ composite particles could prevent the recombination between photoelectrons and holes because photoinduced electrons on the BiOI surface would easily transfer to the TiO_2_, leaving the holes on the BiOI valence band [37]. That is why BiOI-TiO_2_ composite particles have better photocatalytic activities than P25 and BIOI + TiO_2_.

## 4. Conclusions

In summary, the results from our studies demonstrated that BiOI-TiO_2_ composite material prepared by microemulsion methods could enhance photocatalytic activity. When the molar ratio of BiOI to TiO_2_ was 75%, the catalysts showed enhancing efficiency for photocatalytic degradation of BPA in comparison with other as-prepared nanoparticles. The degradation was found to follow pseudo-first-order kinetics. The nanoparticles dosage and initial solution pH have significant influence on the photocatalytic degradation kinetics. The study suggests that the BiOI-TiO_2_ composite material is promising visible-light-driven photocatalysts for environmental applications.

## Figures and Tables

**Table 1:** Textural properties of BiOI-TiO_2_ particles.

Samples	ABET (m^2^g^−1^)	VBJH (cm^3^g^−1^)
TiO_2_	193.7	0.3612
25% BiOI-TiO_2_	145.3	0.2768
50% BiOI-TiO_2_	101.1	0.2169
75% BiOI-TiO_2_	51.9	0.1715
100% BiOI-TiO_2_	40.7	0.1361
BiOI	12.2	0.0359

**Table 2:** The degradation kinetic data under different experimental conditions.

Bi/Ti (molar rate)	Dose (g/L)	Initial pH	*k*_obs_ (min^−1^)	*R* ^2^
25%	0.500	5.8	0.097	0.976
50%	0.500	5.8	0.150	0.987
75%	0.500	5.8	0.241	0.993
100%	0.500	5.8	0.039	0.971
75%	0.000	5.8	0.015	0.936
75%	0.333	5.8	0.122	0.998
75%	0.500	5.8	0.241	0.993
75%	0.667	5.8	0.234	0.987
75%	0.500	2.9	0.101	0.993
75%	0.500	5.0	0.146	0.985
75%	0.500	7.1	0.144	0.972
75%	0.500	9.0	0.222	0.997
75%	0.500	10.0	0.101	0.998
